# The impact of rickets on growth and morbidity during recovery among children with complicated severe acute malnutrition in Kenya: A cohort study

**DOI:** 10.1111/mcn.12569

**Published:** 2017-11-27

**Authors:** Moses M. Ngari, Johnstone Thitiri, Laura Mwalekwa, Molline Timbwa, Per Ole Iversen, Greg W. Fegan, James A. Berkley

**Affiliations:** ^1^ KEMRI/Wellcome Trust Research Programme Kilifi Kenya; ^2^ The Childhood Acute Illness and Nutrition Network (CHAIN) Nairobi Kenya; ^3^ Department of Nutrition, IMB University of Oslo Oslo Norway; ^4^ Department of Haematology Oslo University Hospital Oslo Norway; ^5^ Swansea Trials Unit Swansea University Medical School Swansea UK; ^6^ Centre for Tropical Medicine and Global Health University of Oxford Oxford UK

**Keywords:** children, mortality, rickets, severe acute malnutrition, severe pneumonia

## Abstract

The effects of rickets on children recovery from severe acute malnutrition (SAM) are unknown. Rickets may affect both growth and susceptibility to infectious diseases. We investigated the associations of clinically diagnosed rickets with life‐threatening events and anthropometric recovery during 1 year following inpatient treatment for complicated SAM. This was a secondary analysis of clinical trial data among non‐human immunodeficiency virus‐infected Kenyan children with complicated SAM (2–59 months) followed for 1 year posthospital discharge (ClinicalTrials.gov ID NCT00934492). The outcomes were mortality, hospital readmissions, and growth during 12 months. The main exposure was clinically diagnosed rickets at baseline. Of 1,778 children recruited, 230 (12.9%, 95% CI [11.4, 14 .6]) had clinical signs of rickets at baseline. Enrolment at an urban site, height‐for‐age and head circumference‐for‐age *z* scores were associated with rickets. Rickets at study enrolment was associated with increased mortality (adjusted Hazard Ratio [aHR] 1.61, 95% CI [1.14, 2.27]), any readmission (aHR 1.37, 95% CI [1.09, 1.72]), readmission for severe pneumonia (aHR 1.37, 95% CI [1.05, 1.79]), but not readmission with diarrhoea (aHR 1.05, 95% CI [0.73, 1.51]). Rickets was associated with increased height gain (centimetres), adjusted regression coefficient 0.19 (95% CI [0.10, 0.28]), but not changes in head circumference, mid‐upper arm circumference, or weight. Rickets was common among children with SAM at urban sites and associated with increased risks of severe pneumonia and death. Increased height gain may have resulted from vitamin D and calcium treatment. Future work should explore possibility of other concurrent micronutrient deficiencies and optimal treatment of rickets in this high‐risk population.

## 1 BACKGROUND

Rickets in children is a disease characterized by inadequate or defective bone mineralization and disruption of growth plates caused by deficiency of vitamin D or calcium or impaired metabolism of phosphate (Munns et al., 2016; Razali, Hwu, & Thilakavathy, 2015). It manifests as skeletal abnormalities, including bowed legs, widening wrist, craniotabes (abnormal softening of skull), rachitic rosary (enlarged costochondral junctions of ribs resembling rosary beads), and frontal bossing (prominent forehead; Fukumoto et al., 2015). In developed countries, rickets appears to be re‐emerging after decades of low prevalence (Robinson et al., 2006). Rickets is often considered a rare disease in tropical regions because of the role of sunlight in synthesis of vitamin D (Lerch & Meissner, 2007). However, there is evidence it is prevalent in countries with abundant sunlight (Al‐Atawi, Al‐Alwan, Al‐Mutair, Tamim, & Al‐Jurayyan, 2009; Bener & Hoffmann, 2010; Jones et al., 2017; Ozkan, 2010; Wondale, Shiferaw, & Lulseged, 2005). In Kenya, rickets is common in the urban informal settlements of Nairobi and is predominantly caused by vitamin D deficiency (Edwards et al., 2014; Jones et al., 2017).

Apart from its role in bone mineralization, vitamin D plays a vital role in regulating immune responses to infection (Chun, Adams, & Hewison, 2011; Lang & Aspinall, 2017; Rosen, Daich, Soliman, Brathwaite, & Shoenfeld, 2016). Available evidence suggests an association between vitamin D deficiency and risk of pneumonia, acute lower respiratory tract infections, and diarrhoea among children and mortality among adults (Haider, Nagi, & Khan, 2010; Holter et al., 2016; Muhe, Lulseged, Mason, & Simoes, 1997; Thornton, Marin, Mora‐Plazas, & Villamor, 2013; Wayse, Yousafzai, Mogale, & Filteau, 2004). Among children, vitamin D deficiency is associated with growth retardation (Ganmaa et al., 2017). However, there is unclear evidence from interventional trials of vitamin D supplementation concerning reduction of risks of pneumonia, diarrhoea, malaria, hospital readmission, or alterations in growth (Manaseki‐Holland et al., 2012; Smyth & Broderick, 1997; Sudfeld et al., 2017; Yakoob, Salam, Khan, & Bhutta, 2016).

Children with complicated severe acute malnutrition (SAM) remain susceptible to serious infections during recovery (Berkley et al., 2016), and to our knowledge, no study has evaluated the effect of the presence of rickets on their recovery. In this study, we aimed to investigate the associations of clinically diagnosed rickets at study enrolment with anthropometric recovery and life‐threatening events during 1 year following inpatient treatment for complicated SAM.

1.1

 

**Key messages**

- Clinically defined rickets was present among 12.9% children with complicated severe acute malnutrition mostly from urban areas in Kenya.
- Rickets at study enrolment was associated with increased height gain, likely because of the calcium and vitamin D treatment provided.
- Despite treatment, children with rickets at study enrolment remained more susceptible to death and hospital readmission with severe pneumonia compared to children without rickets.

## 2 METHODS AND MATERIALS

### 2.1 Study design

This was a secondary analysis of data from a double blind randomized controlled clinical trial. Data from both the intervention and placebo arms were included in this secondary analysis. The parent trial investigated the efficacy of daily co‐trimoxazole prophylaxis in reducing 1 year postdischarge mortality among severely malnourished children without human immunodeficiency virus (HIV) infection admitted to four hospitals in Kenya (ClinicalTrials.gov ID NCT00934492), as detailed elsewhere (Berkley et al., 2016). In the parent trial, there was no overall effect of daily co‐trimoxazole prophylaxis on mortality or growth (Berkley et al., 2016).

### 2.2 Study setting

The trial recruited 1,778 children from paediatric wards of four hospitals in Kenya from November 2009 to March 2014. Three of the four hospitals were along the Kenyan coastline: Coast General Hospital in Mombasa (a tertiary level hospital), Kilifi County Hospital and Malindi sub‐county hospital, and Mbagathi sub‐county hospital in Nairobi, predominantly serving a slum‐dwelling population. Coast General and Mbagathi sub‐county hospitals were classified as urban hospitals whereas Kilifi and Malindi county hospitals were classified as rural.

Inpatient management and referral of SAM cases to community‐based therapeutic feeding programmes upon discharge followed the Kenya national recommendations and World Health Organization (WHO) guidelines (WHO, 2005). Children with signs of rickets were treated with vitamin D (150,000 IU STAT intramuscular injection for children younger than 6 months and 300,000 IU for children older than 6 months) and calcium supplements (Purecal/Calcimax® 50–75 mg/kg per day for 3 months). The caregivers were advised to expose the children to sunlight for at least 30 min per day. Children receiving rickets treatment were reviewed after 2 weeks to ensure treatment was continuing, as well as during the planned monthly study follow‐up visits for the first 6 months, then bimonthly up to Month 12. If rickets was unresolved or there was minimal improvement after 3 months, the doses were repeated.

### 2.3 Study participants

All children admitted in the paediatric wards of the four hospitals during the study period were screened for SAM, and those eligible and consented were enrolled after completing the stabilization phase of complicated SAM inpatient treatment (WHO, 2013). Children were eligible for the trial if they had a negative HIV rapid‐antibody test, were aged 2 to 59 months, and were hospitalized with complicated SAM defined as mid‐upper arm circumference (MUAC) < 11.5 cm for children 6 to 59 months old or MUAC < 11.0 cm for children 2 to 6 months or oedema at any age (Berkley et al., 2016).

### 2.4 Outcomes

The main outcomes were episodes of life‐threatening events and anthropometry (MUAC, weight, length/height, and head circumference) during the course of the primary trial of 12 months. Life‐threatening events were defined as all‐cause mortality or all hospital readmissions. We also examined readmissions with severe pneumonia or diarrhoea separately. Severe pneumonia was defined using WHO 2015 guidelines as presence of cough or breathing difficulty with either central cyanosis/oxygen saturation < 90% or general danger signs (impaired consciousness/lethargy/inability to breastfeed or drink) or severe respiratory distress (chest indrawing/grunting; WHO, 2005). Diarrhoea was defined as at least three episodes of loose/watery stools in 24 hr requiring hospitalization following WHO 2015 guidelines (WHO, 2005). Anthropometric recovery was defined by changes from the baseline values of absolute MUAC, and *z* scores for weight‐for‐height/length (WHZ), weight‐for‐age (WAZ), height/length‐for‐age (HAZ), and head circumference (HCZ) calculated using 2006 WHO growth references (WHO, 2011).

### 2.5 Exposures

The main exposure of interest was clinically defined rickets at baseline, which was systematically collected as present or not on the trial baseline case report form. A clinical diagnosis of rickets was made by trained study clinicians at study enrolment by the presence of swelling of wrists and ankles, bowed legs, rachitic rosary, craniotabes, or features of rickets on wrist X‐ray. Other baseline exposures examined were age, gender, randomization arm, recruitment site, weight‐for‐height, height‐for‐age, weight‐for‐age, head circumference‐for‐age *z* scores, oedema, and admission diagnosis.

### 2.6 Data sources/measurements

At the time of recruitment to the trial, child demographics, anthropometry, and admission diagnosis were recorded on structured case report forms. During each scheduled visit, anthropometry was performed, and history of readmission to non‐study hospital or treatment as outpatient was documented.

Children weight in kilograms were measured using an electronic scale (Seca 825, Birmingham, UK). Infantometer (Seca 416, Birmingham, UK) was used to measure length and stadiometer (Seca 215, Birmingham, UK) to measure height in centimetres. Standard insertion tape (TALC, St. Albans, UK) was used to measure MUAC in centimetres.

Free walk‐in clinics for unscheduled visits were provided at all the study hospitals where minor illness was treated as outpatient, and children with serious infections were admitted to study hospitals. Readmission diagnosis and causes of deaths were documented by trained study clinicians directly from study hospital records. For any readmission to non‐study hospitals, admission diagnosis was extracted from the hospital documents.

For community deaths, or those at non‐study hospitals where deceased child medical records were not accessible, verbal autopsies were undertaken. Causes of all deaths were assigned by two paediatricians not involved in the trial using participant medical records or verbal autopsies.

### 2.7 Ethics

The Kenya National Ethical Review Committee (SSC 1562) and Oxford Tropical Research Ethics Committee (reference number 18‐09) approved the primary trial and this secondary analysis.

### 2.8 Statistical methods

Children were stratified as either having diagnosis of clinical rickets or not at baseline. To identify factors associated with rickets at baseline, we used multivariable logistic regression analysis, retaining all the variables examined in the univariable model. For analysis of all‐cause mortality, we used single event survival analysis with time at risk defined as the period from the date of enrolment in the study up to the date of death, lost to follow‐up, withdrew, or completed study follow‐up (Month 12). For readmissions to hospital, we performed multiple event survival analysis, allowing participants to contribute more than one event during the follow‐up. Time at risk was period from time of enrolment to the trial to an episode of readmission or death or lost to follow‐up or withdrew or completed study follow‐ups.

Time to any life‐threatening event (all‐cause mortality, all readmissions, readmissions with severe pneumonia or diarrhoea) curves were fitted using the Kaplan–Meier method and log‐rank test used to compare distribution of events between children with and without rickets at study enrolment. The effect of rickets on risk of the life‐threatening events was estimated using Cox proportional regression analysis and reported as hazard ratios (HR) and respective 95% confidence intervals. We performed univariable analysis with the main exposure (baseline rickets) as the only independent variable to yield the crude HR and added a priori risk factors (baseline age, randomization arm, and gender) plus factors associated with baseline rickets to obtain adjusted HR. To test for effect modification, we compared models with and without interaction terms using likelihood‐ratio tests.

To examine effects of rickets on growth, we used generalized estimating equations (GEE) for regression models with exchangeable correlation structure and the respective changes in monthly anthropometry as the dependent variable. We constructed univariable GEE regression models with baseline rickets as the only independent variable to yield the crude regression coefficient. Then, in multivariable GEE regression models, we adjusted for potential confounders: age, gender, urban/rural site, and randomization arm.

For a sensitivity analysis, missing anthropometry from missed visits, or where follow‐up was done at home and only MUAC was taken, were imputed using the “linear interpolation” approach and used in the GEE regression analysis. The method linearly interpolates a missing value by using values before and after the missing one (Engels & Diehr, 2003). This way, we only imputed anthropometry for missed follow‐up; for example, if a child had anthropometry measurement at Visits 1 and 3, but missed anthropometry at Visit 2, Visits 1 and 3 measurements were used to impute value for Visit 2.

No formal sample size estimation was done for this secondary analysis because data from all the children enrolled in primary trial were analysed. The primary trial enrolled 1,778 children, which was adequate to detect one‐third mortality reduction from 15% among the control group with a power of 90% assuming *p* < .05.

All statistical analysis was performed using stata version 13.1 (stataCorp, College Station, TX, USA).

## 3 RESULTS

### 3.1 Participants

Of the 1,778 children recruited in the trial, 230 (12.9%, 95% CI [11.4, 14.6]) had clinical signs of rickets. The prevalence of rickets was higher at urban than rural sites (15% vs. 4.7%, *p* < .001; Table S1). At Month 3 follow‐up assessment, 79/230 (34%) children still had signs of rickets. Children with rickets were younger than those without rickets: median (interquartile range) age 9 (6 to 13) and 11 (7 to 17) months, respectively (*p* = .002). Children with signs of rickets had lower MUAC, WHZ, and HAZ than those without but had larger HCZ and a lower prevalence of oedema (Table 1).

**Table 1:** Selected baseline characteristics of the study participants

Characteristic^a^		Rickets status at baseline		
	Total (*N* = 1,778)	No rickets (*N* = 1,548)	Rickets (*N* = 230)	*p*‐value
Demographics				
Age in months				
<6 months	305 (17.2)	264 (17.1)	41 (17.8)	.002
6 to 11 months	679 (38.2)	573 (37.0)	106 (46.1)	
12 to 23 months	595 (33.5)	523 (33.8)	72 (31.3)	
≥24 months	199 (11.2)	188 (12.1)	11 (4.8)	
Gender (female)	875 (49.2)	766 (49.5)	109 (47.4)	.55
Urban hospitals	1356 (76.3)	1146 (74.0)	210 (91.3)	<.001
Randomized to co‐trimoxazole prophylaxis	887 (49.9)	775 (50.1)	112 (48.7)	.70
Nutritional status				
Nutritional oedema	300 (16.9)	283 (18.3)	17 (7.4)	<.001
Mid‐upper arm circumference (cm), mean ± *SD*	10.6 ± 1.1	10.6 ± 1.1	10.3 ± 1.0	<.001
Weight‐for‐height *z* score, mean ± *SD*	−3.3 ± 1.3	−3.3 ± 1.3	−3.5 ± 1.3	.01
Weight‐for‐age *z* score, mean ± *SD*	−4.0 ± 1.1	−3.9 ± 1.1	−4.3 ± 1.0	<.001
Length‐for‐age *z* score, mean ± *SD*	−2.9 ± 1.7	−2.8 ± 1.6	−3.1 ± 1.7	.02
Head circumference‐for‐age *z* score^b^, mean ± *SD*	−1.8 ± 1.4	−1.8 ± 1.4	−1.4 ± 1.4	.009
Haemoglobin (g/dl)^c^, mean ± *SD*	9.8 ± 2.2	9.7 ± 2.2	10.2 ± 2.2	.001
Signs of vitamin A deficiency	4 (0.2)	3 (0.2)	1 (0.4)	.43
Breastfeeding	1092 (61.4)	918 (59.3)	174 (75.7)	<.001
Admission diagnosis				
Known tuberculosis at enrolment	67 (3.8)	50 (3.2)	17 (7.4)	.002
Index admission for severe pneumonia	656 (36.9)	528 (34.1)	128 (55.7)	<.001
Index admission for diarrhoea	1021 (57.4)	891 (57.6)	130 (56.5)	.77
Treated for shock during index admission	184 (10.4)	154 (10.0)	30 (13.0)	.15

*Note*. *SD* = standard deviation.

a Values presented are *n* (%) unless specified.

b Data collected from April 2011 (*N* = 708).

c Fifty‐two records missing (*N* = 1,726); blood samples were not collected; means were compared using *z* test and proportions using chi‐square test. Vitamin A deficiency was detected using Bitot's spots, dry conjunctiva/cornea, corneal ulceration, or keratomalacia.

### 3.2 Associations with rickets

Enrolment at an urban site, HAZ and HCZ were associated with rickets (Table 2). Breastfeeding, known tuberculosis at enrolment, and an index admission diagnosis of severe pneumonia were associated with signs of rickets in the univariable analysis; however, these associations attenuated in the multivariable analysis (Table 2).

**Table 2:** Univariable and multivariable analysis of factors associated with rickets at baseline

Characteristic	Univariable analysis			Multivariable analysis		
	Crude odds ratios	95% CI	*p*‐value	Adjusted odds ratios	95% CI	*p*‐value
Demographics						
Age (months)	0.97	0.95, 0.99	.001	0.99	0.95, 1.03	.63
Gender (female)	0.92	0.70, 1.21	.55	0.59	0.37, 1.03	.05
Urban hospitals	3.68	2.30, 5.91	<.001	9.32	2.79, 31.13	<.001
Randomized to co‐trimoxazole prophylaxis	0.95	0.72, 1.25	.70	1.02	0.65, 1.58	.95
Nutritional status						
Nutritional oedema	0.36	0.21, 0.59	<.001	0.84	0.32, 2.20	.73
Mid‐upper arm circumference (cm)	0.75	0.66, 0.85	<.001	0.87	0.66, 1.15	.32
Length‐for‐age *z* score	0.91	0.83, 0.99	.02	0.66	0.55, 0.80	<.001
Head circumference‐for‐age *z* score^a^	1.22	1.05, 1.43	.009	1.53	1.24, 1.89	<.001
Haemoglobin (g/dl)^b^	1.10	1.04, 1.17	.001	1.09	0.97, 1.21	.14
Breastfeeding	2.13	1.55, 2.93	<.001	1.88	0.99, 3.55	.05
Admission diagnosis						
Known tuberculosis at enrolment	2.39	1.35, 4.22	.003	1.24	0.37, 4.19	.73
Index admission for severe pneumonia	2.42	1.83, 3.21	<.001	1.31	0.79, 2.16	.30
Index admission for diarrhoea	0.96	0.72, 1.27	.77	0.83	0.50, 1.38	.48
Treated for shock during index admission	1.36	0.89, 2.06	.15	2.10	0.99, 3.97	.05

*Note*. CI = confidence interval.

a Data collected from April 2011 (*N* = 708).

b Fifty‐two records missing (*N* = 1,726) because blood samples were not taken.

### 3.3 Rickets and mortality

**Figure 1:**
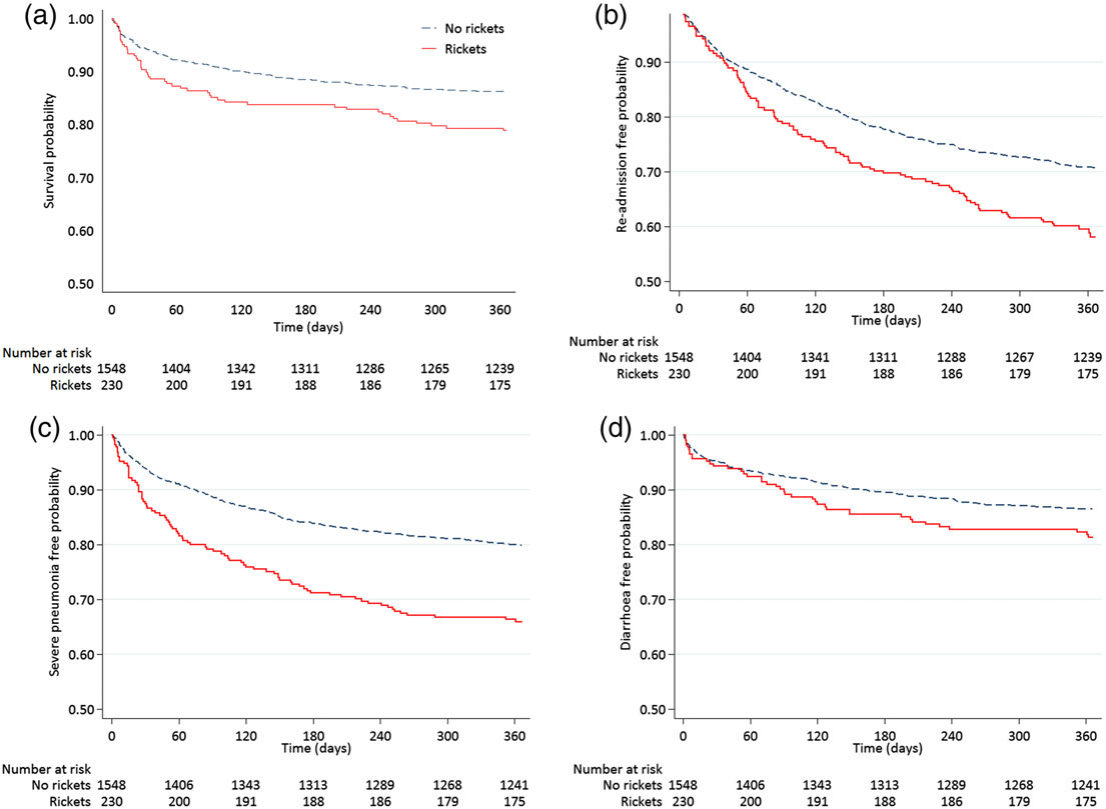
Kaplan–Meier curves for risk of (a) mortality, (b) all readmissions, (c) readmission for severe pneumonia, and (d) readmission for diarrhoea stratified by rickets status at baseline. Blue dashed line = no rickets; red line = rickets

There were 257/1,778 (15%) deaths during 1 year comprising 1518.3 child years of observation (CYO); 48/230 (21%) among children with signs of rickets; and 209/1,548 (14%) among the children without rickets, crude hazard ratio (HR) 1.59 (95% CI [1.16, 2.17]); Table 3 and Figure 1A. After adjusting for potential confounders, rickets at study enrolment was associated with mortality; adjusted HR 1.61 (95% CI [1.14, 2.27]); Table 3. There was no evidence of effect modification by age (*p* = .56), gender (*p* = .63), or site (*p* = .52).

**Table 3:** Univariable and multivariable analysis of events during follow‐up associated with rickets at study enrolment

Type of events	Univariable analysis			Multivariable analysis		
	Crude hazard ratios	95% CI	*p*‐value	Adjusted hazard ratios^a^	95% CI	*p*‐value
Mortality	1.59	1.16, 2.17	.004	1.61	1.14, 2.27	.007
All hospital readmissions	1.55	1.25, 1.91	<.001	1.37	1.09, 1.72	.008
Severe pneumonia	1.87	1.47, 2.24	<.001	1.37	1.05, 1.79	.02
Diarrhoea	1.41	1.00, 1.97	.05	1.05	0.73, 1.51	.77

*Note*. CI = confidence interval.

a Adjusted for gender, age, co‐trimoxazole randomization arm, recruitment site, baseline absolute mid‐upper arm circumference, oedema, and height‐for‐age *z* scores.

### 3.4 Rickets and hospital readmissions

There were 577 hospital readmissions among 425/1,778 (24%) children (Table S2). Rates of readmission among children with and without clinical rickets were 55 (95% CI [46, 67]) and 35 (95% CI [33, 39]) readmissions per 100 CYO. Having signs of rickets at study enrolment was associated with risk of readmission after adjusting for potential confounders, adjusted HR 1.37 (95% CI [1.09, 1.72]); Figure 1b and Table 3 **.** There was no evidence of effect modification by age (*p* = .42), gender (*p* = .53), or site (*p* = .44).

### 3.5 Rickets and severe pneumonia

In total, 392 readmissions with severe pneumonia were recorded during the 1 year of follow‐up among 308/1,778 (17%) participants (Table S2). There were 83 and 309 episodes among children with and without rickets; incidence rate of 44 (95% CI [35.3, 54.3]) and 23 (95% CI [20.8, 26.0]) severe pneumonia readmissions per 100 CYO, respectively. After adjusting for potential confounders, rickets at study enrolment was associated with risk of readmission with severe pneumonia adjusted HR 1.37 (95% CI [1.05, 1.79]), Figure 1c and Table 3. There was no evidence of effect modification by age (*p* = .51), gender (*p* = .73), or site (*p* = .67).

### 3.6 Rickets and severe diarrhoea

There were 243 readmissions for diarrhoea among 218/1,778 (12%) children (Table S2). There were 41 and 202 episodes among children with and without signs of rickets; incidence rate of 22 (95% CI [15.9, 29.4]); and 15 (95% CI [13.3, 17.5]) diarrhoea readmissions per 100 CYO, respectively. Rickets at study enrolment was not associated with the risk of readmission with diarrhoea after adjusting for potential confounders; adjusted HR 1.05 (95% CI [0.73, 1.51]); Figure 1d and Table 3 **.** There was no evidence of effect modification by age (*p* = .55), gender (*p* = .78), or site (*p* = .34).

### 3.7 Rickets and growth

Clinical signs of rickets at study enrolment were associated with increased height growth; adjusted regression coefficient 0.19 (95% CI [0.10, 0.28]); Table 4 and Figure S1. However, signs of rickets had no effect on head circumference, MUAC, and weight growth (Table 4 and Figure S1).

**Table 4:** Effects of baseline rickets on changes in anthropometry during 12 months follow‐up

	Univariable analysis			Multivariable analysis		
	Crude coefficient	95% CI	*p*‐value	Adjusted coefficient^a^	95% CI	*p*‐value
Monthly change in HAZ	0.16	0.07, 0.24	.001	0.19	0.10, 0.28	<.001
Monthly change in HCAZ	0.02	−0.11, 0.14	.81	0.01	−0.12, 0.13	.93
Monthly change in WAZ	0.03	−0.09, 0.15	.60	0.05	−0.07, 0.17	.40
Monthly change in WHZ	−0.12	−0.28, 0.04	.14	−0.10	−0.26, 0.05	.19
Monthly change in MUAC	−0.09	−0.23, 0.05	.23	−0.05	−0.20, 0.09	.45

*Note*. HAZ = height‐for‐age *z* score; HCAZ = head circumference‐for‐age *z* score; WAZ = weight‐for‐age *z* score, WHZ = weight‐for‐height *z* score; MUAC = mid‐upper arm circumference.

a Adjusted for gender, age, co‐trimoxazole randomization arm, and urban/rural hospital.

Results were similar in a sensitivity analysis with imputation of missing anthropometric data, where signs of rickets were associated with increased height growth; adjusted regression coefficient 0.19 (95% CI [0.10, 0.28]), but not with head circumference, MUAC, or weight gain (Tables S3 and S4).

## 4 DISCUSSION

Clinical signs of rickets were common among Kenyan children who were hospitalized with complicated SAM and of prognostic importance for both mortality and for subsequent episodes of severe pneumonia despite treatment with calcium and vitamin D. Rickets occurred predominantly in urban sites and was associated with stunting. An association with increased head circumference is likely to have been due to the inclusion of craniotabes in the diagnostic criteria. However, the clinical diagnosis of rickets in the absence of systematic measurement of vitamin D, calcium, alkaline phosphatase, and their pathways is necessarily subjective. In the parent trial, the individual signs used to identify rickets were not collected; however, the young age of cases (9 months) indicates that the classical presentation of bowed legs in walking‐age children is not the predominant syndrome, as was also demonstrated in an urban community setting in Nairobi (Jones et al., 2017).

Clinical signs of rickets are suggestive of calcium and/or vitamin D deficiency and have high sensitivity for these conditions (Thacher, Fischer, & Pettifor, 2002). However, they may also reflect a diet deficient in other micronutrients affecting both growth and immunity. Evidence from other studies suggest that deficiency of vitamins A, D, and E can occur concurrently and are associated with risk of respiratory tract infections (Zhang et al., 2016). Children with clinical signs of rickets were more stunted and wasted; however, clinical rickets was associated with risk of mortality and serious infections requiring hospitalization even after adjusting for these anthropometrics measurements. Vitamin D plays a major role in modulating both innate and adaptive immunity (Rosen et al., 2016).

We found that linear growth, but not markers of wasting, was improved in children with signs of rickets at baseline, even after adjustment for age. This was largely accounted for by a smaller decline in height/length‐for‐age *z* score between baseline and Month 1 (Figure S1). The most likely explanation is that these children were treated with calcium and vitamin D additional to that contained in therapeutic milks and ready‐to‐use therapeutic food, and this promoted linear growth. Previous studies have reported that vitamin D supplementation increased height growth (Ganmaa et al., 2017). Another potential explanation is regression to the mean; however, the fact that the height/length‐for‐age *z* scores of children with and without rickets do not converge (Figure S1) strongly suggests otherwise.

The prevalence of clinical rickets in our study agrees with that from India (15.4%) and Ethiopia (10.5%) (Desyibelew, Fekadu, & Woldie, 2017; Kumar, Singh, Joshi, Singh, & Bijesh, 2014). The finding that clinically defined rickets was associated with baseline stunting and wasting agrees with previous reports (H. L. Jones et al., 2015). In our study, clinical rickets cases were predominantly from urban sites as previously reported (Edwards et al., 2014; K. D. J. Jones et al., 2017). Other studies, although not among children hospitalized with complicated SAM, have found evidence of association between vitamin D deficiency and severe pneumonia, gastrointestinal infection, and growth (Ganmaa et al., 2017; Haider et al., 2010; Muhe et al., 1997; Thornton et al., 2013). This partially concurs with our study findings; however, in our population, diarrhoea risk was not associated with clinical defined rickets.

There is inconclusive evidence from prior studies on the efficacy of vitamin D supplementation in reducing all‐cause mortality, hospital admissions, or incidence of severe pneumonia and diarrhoea (Das, Singh, Panigrahi, & Naik, 2013; Yakoob et al., 2016). Our results suggest that the treatment for rickets that we gave did not result in reduction in risks of mortality or of readmission to that of the other children in the trial. We did not measure compliance with rickets treatment; however, there is the possibility that the current rickets treatment guidelines are not adequate to address all the macronutrient demands for children recovering from SAM. Future studies should explore the possibility of concurrent micronutrient deficiency and optimal treatment among this population of children recovering from SAM. Such research should involve screening for multiple macronutrient deficiencies and design of robust clinical trials to evaluate potential interventions.

### 4.1 Strengths and limitations

The main strength of the study was its prospective design that attained a 95% follow‐up for 1 year and the well documented life‐threatening events.

The major limitation was the use of clinical signs to diagnose rickets. Although clinical signs are sensitive in identification of rickets cases, they have poor specificity, and therefore, we could have missed some cases especially among children with oedema that could blur the clinical signs (Jones et al., 2017; Thacher et al., 2002), that is, many other children may have had low calcium or vitamin D, and this may have introduced bias. Severe pneumonia and diarrhoea were defined using the WHO clinical signs; although these signs are sensitive, they have low specificity. However, this reflects the diagnostic criteria commonly used in such poor‐resource settings and do not preclude the fact that children diagnosed with clinical signs of rickets had higher risks of life‐threatening infections requiring hospital admission or causing death.

This being a secondary data analysis of a clinical trial, active follow‐ups and the free walk‐in clinics offered could have led to overidentification of serious infections leading to over‐reporting of readmissions compared to children in the community. It is also possible, the non‐severe illness treated during the follow‐up visits could have prevented them progressing to severe infection requiring readmission. Missing follow‐up anthropometry could have introduced bias, but imputing missing data did not change our results significantly. Our results should be interpreted cautiously because it is not generalizable to children with uncomplicated SAM or complicated SAM infected with HIV.

## 5 CONCLUSION

Rickets is common among children with complicated SAM, predominantly from urban sites, and is associated with increased risk of death and hospital admissions with severe pneumonia. Increased height growth may have been due to the calcium and vitamin D treatment provided. Future work should explore the possibility of other micronutrient deficiency and optimal treatment of rickets in this high‐risk population.

## CONFLICTS OF INTEREST

The authors declare that they have no conflicts of interest.

## CONTRIBUTIONS

MMN designed the study, performed statistical analysis, and writing of the first manuscript draft. POI and GF provided supervision and advice on design, analysis, and interpretation. JT, LM and MT were responsible for clinical care and data collection. JAB was the principal investigator of the parent trial and offered overall supervision in design, analysis, and interpretation of the study results. All authors reviewed and agreed on the final manuscript.

## Supporting information

Table S1: Distribution of rickets across the recruitment sites.
Table S2: Distribution of Life‐threatening events during one year follow‐up.
Table S3: Effects of baseline rickets on changes in anthropometry during 12 months follow‐up using imputed data for missing values.
Table S4: Number of missing monthly anthropometry records during follow up that were imputed.
Figure S1: Changes in anthropometry A; HAZ, B; HCZ, C; MUAC and D; WAZ stratified by rickets status at baseline.
