# Supplementary material for: The impact of rickets on growth and morbidity during recovery among children with complicated severe acute malnutrition in Kenya: A cohort study
Source: Matern Child Nutr. 2017 Nov 27;14(2):e12569. doi: 10.1111/mcn.12569 (PMC5901410; doi:10.1111/mcn.12569)
Supplement: Supplementary file 1 — Table S1: Distribution of rickets across the recruitment sites. Table S2: Distribution of Life‐threatening events during one year follow‐up. Table S3: Effects of baseline rickets on changes in anthropometry during 12 months follow‐up using imputed data for missing values. Table S4: Number of missing monthly anthropometry records during follow up that were imputed. Figure S1: Changes in anthropometry A; HAZ, B; HCZ, C; MUAC and D; WAZ stratified by rickets status at baseline. [file MCN-14-e12569-s001.docx]

**The impact of rickets on growth and morbidity during recovery among children with complicated severe acute malnutrition in Kenya - a cohort study.**

**Supplementary Material**

Table of Content

[Supplementary Table 1: Distribution of rickets across the recruitment sites. 2](#_Toc491334619)

[Supplementary Table 2: Distribution of Life-threatening events during one year follow-up. 2](#_Toc491334620)

[Supplementary Figure 1: Changes in anthropometry A; HAZ, B; HCZ, C; MUAC and D; WAZ stratified by rickets status at baseline. 3](#_Toc491334621)

[Supplementary Table 3: Effects of baseline rickets on changes in anthropometry during 12 months follow-up using imputed data for missing values. 4](#_Toc491334622)

[Supplementary Table 4: Number of missing monthly anthropometry records during follow up that were imputed. 5](#_Toc491334623)

| **Recruitment site** | **Total recruited** | **Signs of rickets** | **Proportion (%)** |
| --- | --- | --- | --- |
| Kilifi County Hospital | 151 | 1 | 0.7 |
| Malindi sub-county Hospital | 271 | 19 | 7.0 |
| Coast General Hospital (Mombasa)# | 849 | 55 | 6.5 |
| Mbagathi sub-county (Nairobi)# | 507 | 155 | 31 |
| Total | 1778 | 230 | 13 |
| #-the two sites were classified as urban sites. **Supplementary Table 1**: Distribution of rickets across the recruitment sites. | | | |

|  | **Signs of rickets/none** | **Total events** | **Episode one events** | **Episode two events** | **Episode three events** | **Episode four events** | **Episode ≥5 events** |
| --- | --- | --- | --- | --- | --- | --- | --- |
| All-cause mortality | Signs of rickets (N=230) | 48 (21%) |  |  |  |  |  |
|  | No rickets signs (N=1548) | 209 (14%) |  |  |  |  |  |
| All admissions | Signs of rickets (N=230) | 105 | 76 | 25 | 3 | 1 |  |
|  | No rickets signs (N=1548) | 472 | 349 | 84 | 25 | 9 | 5 |
| Admissions with severe pneumonia | Signs of rickets (N=230) | 83 | 69 | 11 | 2 | 1 |  |
|  | No rickets signs (N=1548) | 309 | 239 | 50 | 13 | 7 |  |
| Admissions with diarrhoea | Signs of rickets (N=230) | 41 | 38 | 3 |  |  |  |
|  | No rickets signs (N=1548) | 202 | 180 | 20 | 2 |  |  |
| A child could be re-admitted with more than one diagnosis. **Supplementary Table 2**: Distribution of Life-threatening events during one year follow-up. | | | | | | | |

| **A**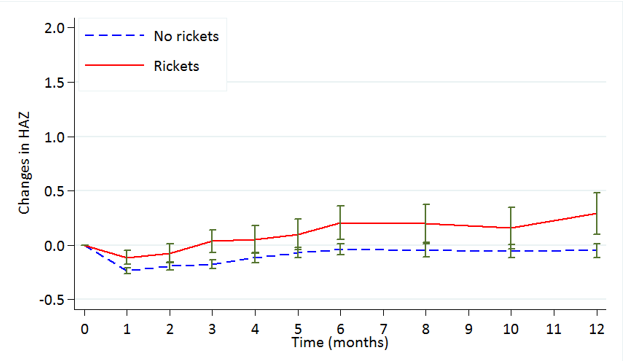 | **B**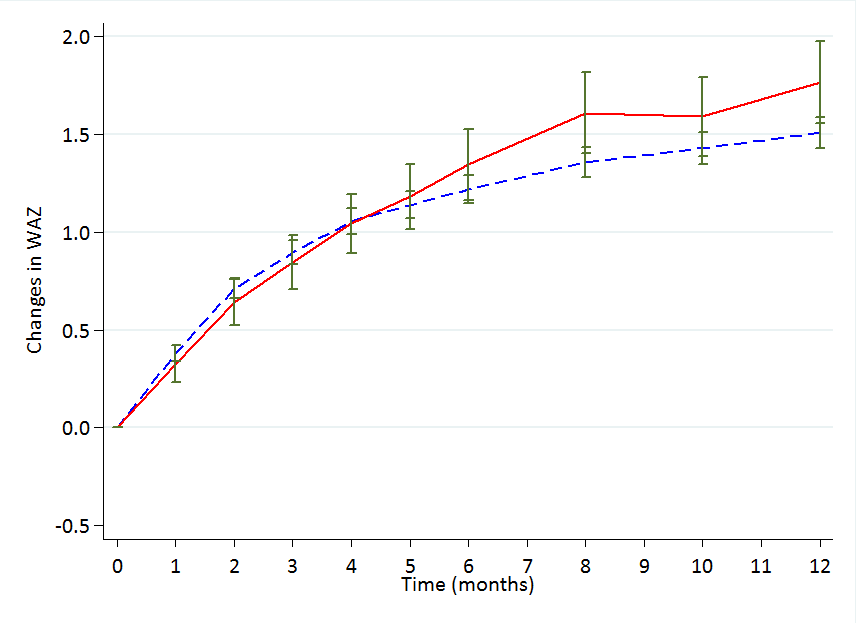 |
| --- | --- |
| **C**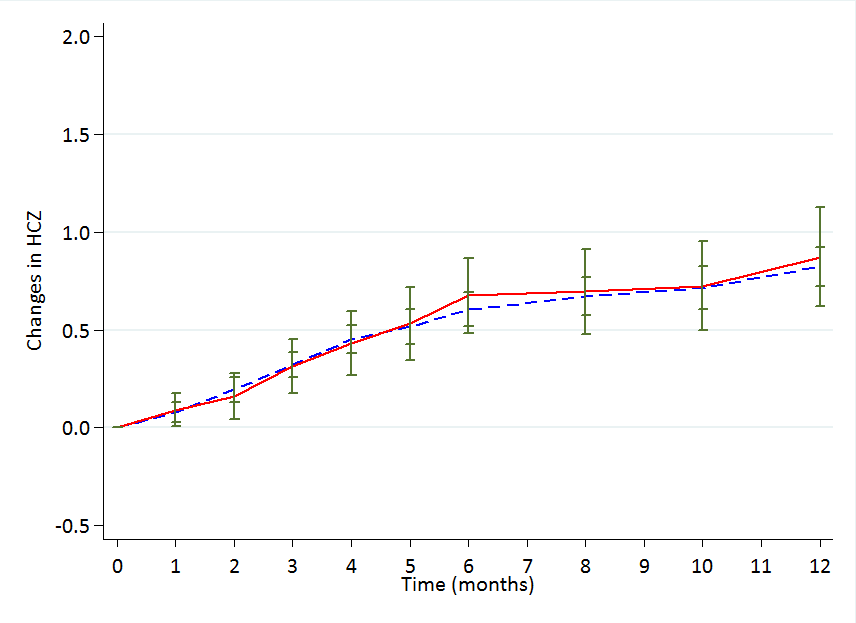 | **D**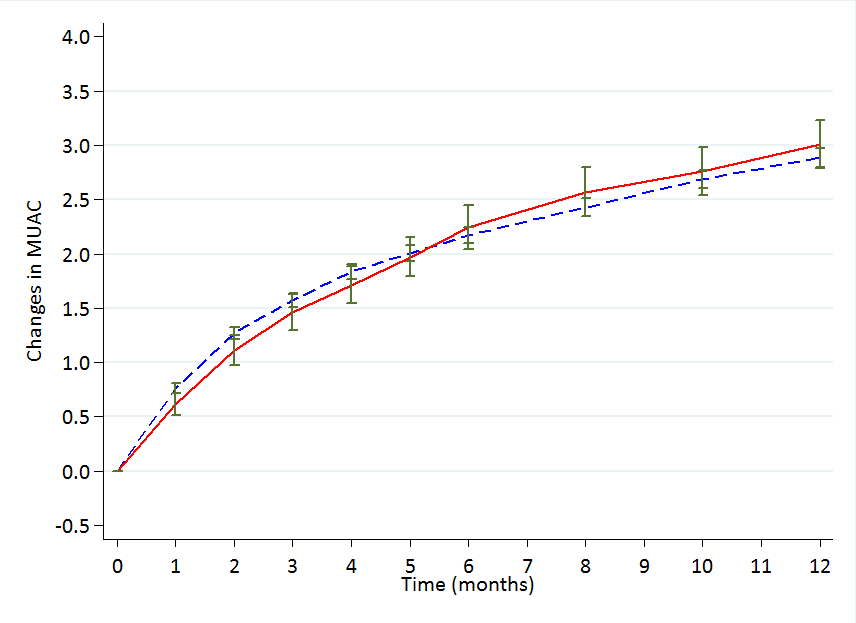 |
| Data are means, error bars are 95% CI, HAZ-Height-for-age, HCZ-Head circumference-for-age and WAZ-Weight-for-age z-scores, MUAC-Mid-upper arm circumference (cm), dashed blue line-no rickets and red line-rickets **Supplementary Figure 1**: Changes in anthropometry **A**; HAZ, **B;** HCZ, **C**; MUAC and **D;** WAZ stratified by rickets status at baseline. | |

|  | Univariate analysis | | | Multivariate analysis | | |
| --- | --- | --- | --- | --- | --- | --- |
|  | Crude Coefficient | 95% CI | P-value | Adjusted Coefficient* | 95% CI | P-value |
| Monthly change in HAZ | 0.16 | 0.07 to 0.25 | <0.001 | 0.19 | 0.10 to 0.28 | <0.001 |
| Monthly change in HCAZ | 0.02 | -0.11 to 0.14 | 0.80 | 0.01 | -0.12 to 0.13 | 0.92 |
| Monthly change in WAZ | 0.03 | -0.09 to 0.15 | 0.57 | 0.05 | -0.07 to 0.17 | 0.39 |
| Monthly change in WHZ | -0.12 | -0.28 to 0.04 | 0.14 | -0.11 | -0.26 to 0.05 | 0.19 |
| Monthly change in MUAC | -0.09 | -0.23 to 0.06 | 0.23 | -0.05 | -0.20 to 0.09 | 0.45 |
| * Adjusted for gender, age, co-trimoxazole randomization arm and urban/rural hospital, HAZ- height-for-age z-score, HCAZ- head circumference-for-age z-score, WAZ- weight-for-age z-score, WHZ- weight-for-height z-score and MUAC-Mid upper arm circumference Supplementary Table 3: Effects of baseline rickets on changes in anthropometry during 12 months follow-up using imputed data for missing values. | | | | | | |

| **Characteristics** | **Baseline** | **M1** | **M2** | **M3** | **M4** | **M5** | **M6** | **M8** | **M10** | **M12** |
| --- | --- | --- | --- | --- | --- | --- | --- | --- | --- | --- |
| Participants in  follow-up- N | 1778 | 1643 | 1591 | 1549 | 1521 | 1494 | 1493 | 1462 | 1439 | 1431 |
| MUAC | - | 24 | 18 | 22 | 33 | 45 | 18 | 46 | 65 | - |
| WHZ | - | 49 | 20 | 43 | 49 | 62 | 15 | 62 | 85 | - |
| WAZ | - | 46 | 20 | 39 | 46 | 60 | 15 | 63 | 85 | - |
| HAZ | - | 49 | 17 | 44 | 47 | 61 | 15 | 58 | 83 | - |
| HCZ | - | 11 | 8 | 18 | 22 | 30 | 8 | 31 | 56 | - |
| MUAC-Mid-upper arm circumference (cm), WHZ- weight-for-height z-score, WAZ weight-for-age z-score, HAZ- height-for-age z-score, HCZ- head circumference-for-age z-score, no imputation was done at baseline and at month 12 because there were no subsequent measurements before and after. **Supplementary Table 4:** Number of missing monthly anthropometry records during follow up that were imputed. | | | | | | | | | | |
